# Evaluating Adjuvant Radiation Therapy Survival Benefit in Early-Stage HER2-Positive Invasive Breast Cancer Following Breast-Conserving Surgery: A National Cohort Aligned with NRG-BR008 HERO Trial

**DOI:** 10.3390/cancers18030352

**Published:** 2026-01-23

**Authors:** Jonathon S. Cummock, Ali J. Haider, Mohummad Kazmi, Waqar M. Haque, Andrew M. Farach, E. Brian Butler, Bin S. Teh

**Affiliations:** 1 Department of Medical Sciences, Texas A&M University Naresh K. Vashisht College of Medicine, Bryan, TX 77807, USA; jonnycummock@tamu.edu; 2 Department of Radiation Oncology, University of Texas Medical Branch, Galveston, TX 77550, USA; alhaider@utmb.edu; 3 University of North Texas Health Science Center, Texas College of Osteopathic Medicine, Fort Worth, TX 76137, USA; mohummadkazmi@my.unthsc.edu; 4 Department of Radiation Oncology, Houston Methodist Hospital, Houston, TX 77030, USA; wmhaque@houstonmethodist.org (W.M.H.); amfarach@houstonmethodist.org (A.M.F.); ebutler@houstonmethodist.org (E.B.B.)

**Keywords:** breast cancer, HER2-positive carcinoma, adjuvant radiotherapy, neoadjuvant therapy, breast-conserving surgery, survival analysis, pathologic complete response, propensity score, cohort studies, databases

## Abstract

**Background and purpose:** The role of adjuvant radiation therapy (RT) in early-stage HER2-positive breast cancer treated with breast-conserving surgery (BCS) and systemic therapy remains uncertain in the era of HER2-targeted regimens. This study evaluates the survival impact of RT in patients aligned with the HERO RT de-escalation trial (NRG-BR008). **Materials and methods:** We queried the National Cancer Database for patients with early-stage HER2-positive invasive breast carcinoma treated with BCS and systemic therapy, stratified into HERO trial-aligned cohorts: Arm 1 (adjuvant systemic therapy) vs. Arm 2 (neoadjuvant systemic therapy, pathologic complete response). Within each cohort, patients receiving adjuvant RT were compared with those omitting RT. In the primary analysis, patients were propensity score matched (PSM) on demographics, diagnosis years, tumor characteristics, and trial stratification variables. Inverse probability of treatment weighting (IPTW) was additionally performed as a sensitivity analysis. Overall survival was evaluated using Kaplan–Meier, Cox regression, and restricted mean survival time (RMST). **Results:** In **Arm 1** (818 patients, 94 deaths), 5-year OS was 96.9% with RT vs. 88.0% without RT, and 10-year OS was 94.3% vs. 68.5% (log-rank *p* < 0.001). RT omission was associated with higher mortality in the PSM Cox model (HR, 4.78; 95% CI, 2.84–8.02; *p* < 0.001), with an RMST advantage favoring RT of +2.86 months at 5 years and +12.55 months at 10 years (*p* < 0.001). In Arm 2 (176 patients, 10 deaths), 5-year OS was 97.6% with RT vs. 91.1% without RT, and OS at 107 months was 94.8% vs. 91.1% (log-rank *p* = 0.13). RT omission was not statistically significant in the PSM Cox model (HR, 3.40; 95% CI, 0.82–14.05; *p* = 0.09), though RMST favored RT (+1.83 months at 5 years, *p* = 0.004; +3.91 months at 107 months, *p* = 0.03). IPTW analyses were directionally consistent in Arm 1 (HR, 3.26; 95% CI, 2.52–4.21; *p* < 0.001) and inconclusive in **Arm 2** (HR, 1.78; 95% CI, 0.80–3.95; *p* = 0.16). **Conclusions:** In this HERO-aligned national cohort, RT omission was associated with inferior OS in patients treated with adjuvant systemic therapy after BCS. Findings in the neoadjuvant pCR cohort were imprecise and hypothesis-generating. Given the retrospective registry design, lack of recurrence-specific endpoints, and potential residual confounding, results should not be interpreted as causal but support continued RT use outside prospective de-escalation trials.

## Simple Summary

Radiation therapy (RT) after breast-conserving surgery is standard for invasive breast cancer, but de-escalation is being studied for select low-risk HER2-positive tumors. Using the National Cancer Database, we assembled the following two cohorts aligned with the NRG-BR008 (HERO) trial framework: patients receiving systemic therapy after surgery (adjuvant pathway) and those treated before surgery (neoadjuvant pathway) who achieved a pathologic complete response. We compared overall survival for patients who did versus did not receive postoperative RT using propensity score matching and time-to-event models. RT omission was associated with worse survival in the adjuvant pathway (hazard ratio ~5). In the neoadjuvant pathway, RT was associated with a smaller, statistically non-significant survival difference, consistent with limited events and imprecision. These observational findings support continued RT use outside clinical trials pending prospective de-escalation data.

## 1. Introduction

Breast-conserving surgery (BCS) followed by adjuvant radiation therapy (RT) is a foundational component of local-regional management for early-stage invasive breast cancer, with randomized evidence demonstrating durable reductions in ipsilateral breast events and breast cancer mortality [1,2,3]. Consistent with this evidence, contemporary guidelines recommend adjuvant RT after BCS for early-stage invasive breast cancer, including HER2-positive disease (NCCN [4], ESMO [5]).

In early-stage HER2-positive disease, modern systemic therapy with trastuzumab, pertuzumab, and other HER2-directed agents has dramatically improved systemic disease control and long-term survival, including in patients with small, node-negative (pT1N0) tumors [6,7]. Landmark trials such as APT [6] and ATEMPT [7] have demonstrated exceptionally high invasive disease-free survival (iDFS) rates (>97%) with trastuzumab-based regimens, resulting in favorable long-term outcomes. This therapeutic shift has prompted interest in RT de-escalation strategies; however, whether RT omission can be performed without compromising long-term outcomes remains uncertain for HER2-positive breast cancer.

The recently closed NRG-BR008 (HERO) [8,9,10] is a randomized phase III non-inferiority trial designed to prospectively evaluate RT optimization/omission after BCS in a low-risk HER2-positive population (early-stage) receiving contemporary systemic therapy. While the HERO trial was expected to provide definitive evidence regarding the role of RT in this population, accrual was closed due to insufficient enrollment, leaving a gap in prospective evidence. In this context, large-scale real-world analyses can provide timely, practice-relevant insights to guide decision-making for patients and clinicians. This study uses a large national cohort of patients meeting HERO trial-aligned eligibility criteria, stratified by systemic therapy sequence (adjuvant vs. neoadjuvant pathways), to assess whether adjuvant RT is associated with improved overall survival (OS) in patients with early-stage HER2-positive breast cancer. This analysis incorporates both adjuvant and neoadjuvant systemic therapy cohorts, providing a broader view of RT’s real-world impact across different treatment pathways.

## 2. Materials and Methods

**Data Source and Study Populations:** This retrospective cohort study utilized data from the National Cancer Database (NCDB), a joint project of the Commission on Cancer of the American College of Surgeons and the American Cancer Society. Containing records from over 1500 hospitals with American College of Surgeons-accredited cancer programs, the NCDB captures data on approximately 70% of all newly diagnosed cancers in the United States, including information on patient demographics, tumor characteristics, and survival [11,12]. The NCDB underrepresents rural and minority populations, as it is not population-based [13]. This study utilized de-identified patient data obtained from a participant user file. As this research involved analysis of preexisting, anonymized records with no patient identifiers, it was exempt from institutional review board approval.

**Data Availability:** The data that support the findings of this study are available from the NCDB, but restrictions apply to the availability of these data, which were used under license for the current study, and so are not publicly available. Data are, however, available from the authors upon reasonable request and with permission of the NCDB.

**Patient Selection and Cohorts:** Using the NCDB (2004–2021), we identified patients aged ≥ 40 years with early-stage HER2-positive invasive breast cancer treated with breast-conserving surgery (BCS) and systemic therapy, applying eligibility criteria designed to mirror the NRG-BR008 (HERO) framework [10]. All patients had confirmed pT1-2N0M0 staging with axillary evaluation (sentinel node biopsy or axillary lymph node dissection) and known estrogen receptor (ER) and progesterone receptor (PgR) status.

**Figure 1:**
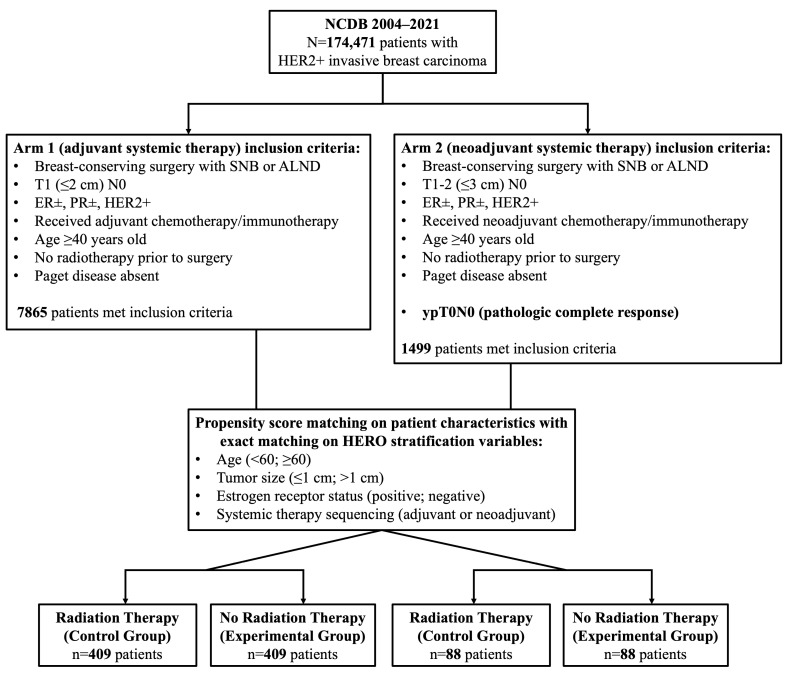
Patient selection flow diagram. Flow diagram illustrating the identification of patients with HER2-positive invasive breast carcinoma from the National Cancer Database (NCDB) and stratification into adjuvant systemic therapy (Arm 1) and neoadjuvant systemic therapy (Arm 2) cohorts. Patients were categorized by receipt of adjuvant radiation therapy (RT vs. no RT). SNB = sentinel lymph node biopsy; ALND = axillary lymph node dissection.

Patients were stratified into two cohorts based on systemic therapy sequencing, in alignment with the HERO trial protocol: Arm 1 (adjuvant systemic therapy cohort) included patients with tumors ≤ 2 cm who received systemic therapy after surgery, and Arm 2 (neoadjuvant systemic therapy cohort) included patients with tumors ≤ 3 cm who received systemic therapy prior to surgery and subsequently achieved pathologic complete response (ypT0N0). In each arm, patients were further categorized based on receipt of radiation therapy. Patients who received RT were designated as the control group, and those who omitted RT were designated as the experimental group; hazard ratios are reported with the RT group as the reference. Patients were excluded if they lacked complete data on tumor size, hormone receptor status, systemic therapy sequence, RT status, or survival time. Figure 1 summarizes the inclusion and stratification process.

**Statistical Procedures:** The primary endpoint was overall survival, defined as time from diagnosis to death from any cause, with patients censored at last contact. To mitigate immortal time bias related to post-diagnosis treatment allocation, survival time was left-truncated at 6 months from diagnosis. Analyses were performed separately within each cohort (Arm 1 and Arm 2).

Propensity scores for receipt of RT were estimated within each cohort using multivariable logistic regression including age, race, Hispanic ethnicity, comorbidity burden (Charlson–Deyo score), insurance status, urbanicity, diagnosis era (modeled as defined in the analytic dataset), tumor grade, and lymphovascular invasion (LVI). To enforce exchangeability within the region of overlap, observations outside the common support of the propensity score distribution between RT and no-RT groups were excluded; propensity scores were then re-estimated after this trimming step.

For the primary analysis, we performed 1:1 nearest-neighbor propensity score matching (PSM) without replacement using a caliper of 0.20 standard deviations of the logit of the propensity score, targeting the average treatment effect among controls (ATC). Matching incorporated exact constraints on the HERO stratification variables (age category ≥ 60 vs. <60 years, tumor size category ≥ 1 cm vs. <1 cm, and ER status positive vs. negative) and Mahalanobis distance on continuous age and Charlson–Deyo comorbidity within the propensity score caliper. Covariate balance was assessed using standardized mean differences (SMD), with |SMD| < 0.10 prespecified as acceptable.

Kaplan–Meier methods were used to estimate OS in the matched cohorts, and groups were compared using the log-rank test. The primary effect estimates were obtained using Cox proportional hazards regression, with RT status as the primary exposure; models additionally included age and Charlson–Deyo score and were stratified by tumor size category and ER status in accordance with the HERO framework. Robust variance estimation clustered on matched pairs was used to account for the matched design. The proportional hazards assumption was evaluated using Schoenfeld residuals.

Restricted mean survival time (RMST) differences (RT − no RT) were estimated using the survRM2 framework at prespecified truncation times of 60 and 120 months for Arm 1. For Arm 2, RMST was evaluated at 60 months and at the maximum common follow-up between groups. Ninety-five percent confidence intervals were obtained using bootstrap resampling (2000 iterations), accounting for the matched design. Post hoc detectability was summarized descriptively using Schoenfeld approximations based on the observed number of deaths and is reported to reflect event sparsity.

As a sensitivity analysis to address residual confounding and to provide an alternative causal estimator, inverse probability of treatment weighting (IPTW) was performed using stabilized weights targeting the average treatment effect (ATE), with weights truncated at the 1st and 99th percentiles and restricted to the region of common propensity score support. Weighted Kaplan–Meier and weighted Cox regression (with robust sandwich standard errors) were then performed; RMST analyses were also repeated under IPTW where applicable. Covariate balance was reassessed after weighting using SMD.

All statistical analyses were conducted using R (version 4.5). Key packages included *MatchIt* (version 4.7.2) for propensity score matching, *cobalt* (version 4.6.1) for balance assessment, *survival* (version 3.8-3) for time-to-event analysis, and *survRM2* (version 1.0-4) for RMST estimation. Analyses adhered to the Strengthening the Reporting of Observational Studies in Epidemiology (STROBE) guidelines.

## 3. Results

**Figure 2:**
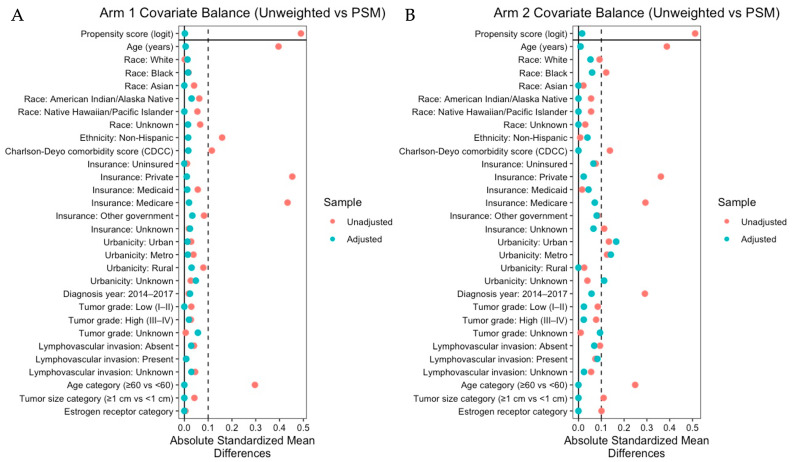
Covariate balance using propensity score matching. Love plots depicting absolute standardized mean differences (ASMDs) for baseline covariates comparing the radiation therapy (RT) and no-RT groups before (unadjusted; red) and after propensity score matching (adjusted; teal). Panel (**A**) shows covariate balance for Arm 1 (adjuvant systemic therapy cohort), and Panel (**B**) shows covariate balance for Arm 2 (neoadjuvant systemic therapy cohort). The vertical dashed line indicates the balance threshold (ASMD = 0.10); values below this threshold are generally interpreted as indicating adequate post-matching balance between treatment groups.

**Cohort Characteristics:** After applying eligibility criteria, restricting to the region of common propensity score support, and left-truncating survival time at 6 months to mitigate immortal time bias, propensity score matching yielded 409 matched pairs (*N* = 818) in Arm 1 (adjuvant systemic therapy) and 88 matched pairs (*N* = 176) in Arm 2 (neoadjuvant systemic therapy, pCR). There were 94 all-cause deaths in Arm 1 and 10 deaths in Arm 2 during follow-up. Baseline characteristics of the matched cohorts are summarized in Table 1. Covariate balance improved substantially after matching, with all measured covariates achieving acceptable standardized mean differences (|SMD| < 0.10) in both arms (Figure 2). In Arm 1, mean age was similar between groups (63.39 vs. 63.45 years), the majority of patients were White (84.1% vs. 84.6%), and comorbidity burden was low (Charlson–Deyo comorbidity index, 0.22 vs. 0.25). Patient diagnosis years ranged from 2007 to 2017, with most patients diagnosed between 2014 and 2017 (80.7% vs. 79.9%). Tumor grade was split evenly between low grade (I–II) and high grade (III–IV), and most tumors presented without lymphovascular invasion (78.5% vs. 77.3%). Exact matching resulted in identical distributions of HERO stratification variables across RT and no-RT groups: age ≥ 60 years (64.5%), tumor size > 1 cm (65.0%), and ER-positive status (77.0%).

In Arm 2, mean age was 60.14 years in the RT group and 60.24 years in the no-RT group, with most patients identifying as White (73.9% vs. 76.1%) and a low mean Charlson–Deyo score (0.23 in both groups). Most patients were diagnosed between 2014 and 2017 (94.3% vs. 95.5%). Tumor grade more often presented as high (61.4% vs. 60.2%), and most tumors presented without lymphovascular invasion (63.6% vs. 60.2%). Exact matching yielded identical distributions of HERO stratification variables across groups: age ≥ 60 years (48.9%), tumor size > 1 cm (94.3%), and ER-positive status (50.0%).

**Figure 3:**
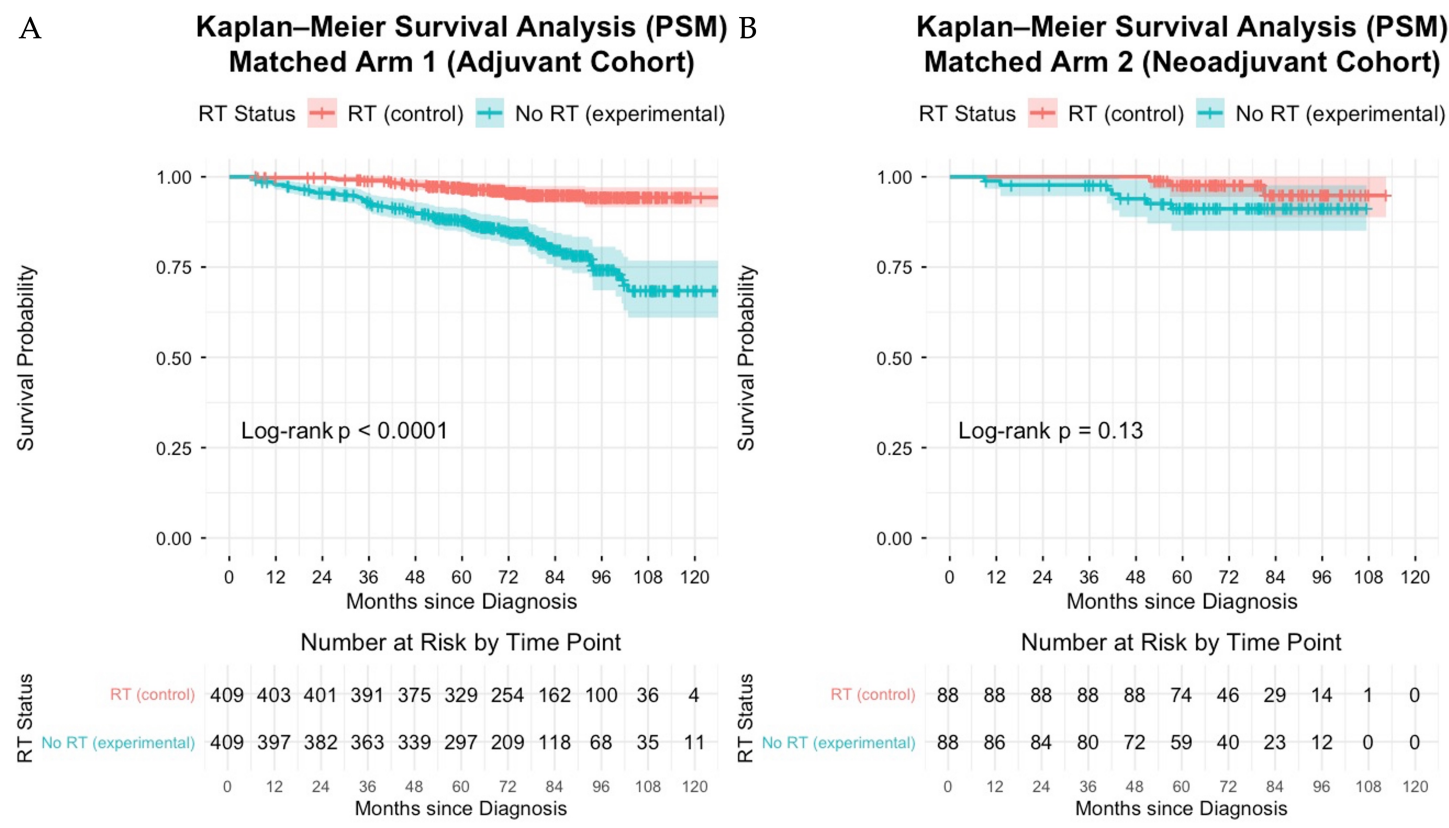
Kaplan–Meier overall survival curves (PSM-matched). Kaplan–Meier estimates of overall survival (OS) comparing propensity-score matched patients treated with adjuvant radiation therapy (RT) vs. no RT, stratified by treatment sequence: Arm 1 (adjuvant systemic therapy cohort), Panel (**A**), and Arm 2 (neoadjuvant systemic therapy cohort), Panel (**B**). Survival time was left-truncated at 6 months to mitigate immortal time bias. *p* values reflect log-rank tests stratified by age, tumor size, and estrogen receptor status. Shaded regions indicate 95% confidence intervals.

**Kaplan–Meier Survival Outcomes:** In Arm 1 (Figure 3A), OS at 5 years was 96.9% (95% CI, 95.2–98.6) in the RT group compared with 88.0% (95% CI, 84.8–91.3) in the no-RT group, and 10-year OS was 94.3% (95% CI, 91.6–97.1) vs. 68.5% (95% CI, 61.0–76.8), respectively (log-rank *p* < 0.001). In Arm 2 (Figure 3B), 5-year OS was 97.6% (95% CI, 94.4–100.0) with RT vs. 91.1% (95% CI, 85.1–97.7) without RT; OS at the maximum common follow-up (107 months) was 94.8% (95% CI, 88.8–100.0) vs. 91.1% (95% CI, 85.1–97.7), respectively (log-rank *p* = 0.13).

**Cox Proportional Hazards Models:** In the matched cohorts, omission of RT (no-RT group) was associated with higher mortality risk in Arm 1 (HR, 4.78; 95% CI, 2.84–8.02; *p* < 0.001) and a similar direction of effect in Arm 2, although with substantial imprecision (HR, 3.40; 95% CI, 0.82–14.05; *p* = 0.09) (Table 2). Models were adjusted for age and Charlson–Deyo comorbidity index and stratified by tumor size category and ER status in accordance with the HERO trial framework. The proportional hazards assumption was not violated for either group (global test *p* = 0.61 and *p* = 0.13, respectively).

**Figure 4:**
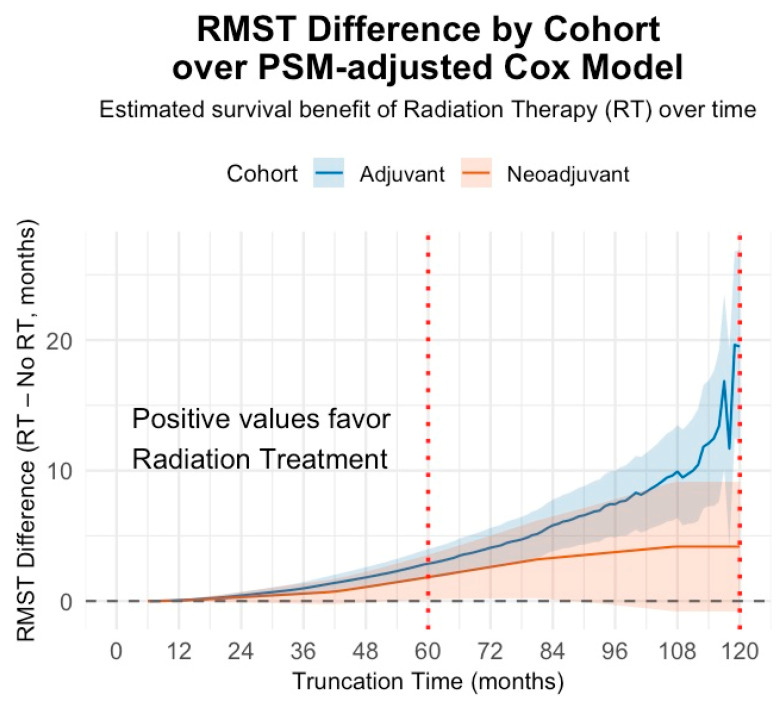
Restricted mean survival time analysis (PSM-matched). RMST difference curves showing the PSM-matched absolute survival benefit of radiation therapy (RT) minus no RT across increasing follow-up durations stratified by treatment sequencing cohort (Arm 1: adjuvant systemic therapy cohort; Arm 2: neoadjuvant systemic therapy cohort). Positive RMST differences indicate a longer average survival time associated with RT. Shaded bands represent 95% confidence intervals; vertical red dotted lines denote clinically relevant follow-up timepoints (5 and 10 years). Survival time was left-truncated at 6 months in both cohorts to mitigate immortal time bias.

**Restricted Mean Survival Time:** RMST analyses demonstrated an adjusted survival benefit associated with RT in both arms (Figure 4). In Arm 1, RMST differences (RT − no RT) were +2.86 months at 60 months (95% CI, 1.95–3.73; *p* < 0.001) and +12.55 months at 120 months (95% CI, 9.85–15.35; *p* < 0.001). In Arm 2, RMST favored RT by +1.83 months at 60 months (95% CI, 0.47–2.97; *p* = 0.004) and +3.91 months at 107 months (95% CI, 0.47–7.26; *p* = 0.03).

**Post Hoc Power and Minimum Detectable Effect:** Post hoc power calculations based on Schoenfeld approximations indicated that, with 94 deaths in cohort 1, the study had limited power to detect modest hazard ratios (power ≈ 25% for HR = 1.30), and an 80% power minimum detectable effect size of approximately HR = 1.78. In cohort 2, only 10 deaths were observed, corresponding to very low power for modest hazard ratios (≈6% for HR = 1.30) and an 80% power minimum detectable effect size of approximately HR = 5.88 (Table 3).

**Sensitivity Analysis (Inverse Probability of Treatment Weighting):** In IPTW analyses targeting the average treatment effect, covariate balance improved after weighting across both cohorts, with the majority of measured baseline covariates achieving acceptable post-weighting standardized mean differences (Supplemental Figure S1). In the IPTW-weighted Kaplan–Meier analyses, overall survival remained consistently lower among patients who omitted RT in the adjuvant cohort, with a highly significant weighted log-rank test (*p* < 0.0001) (Supplemental Figure S2A). In contrast, survival curves in the neoadjuvant cohort demonstrated substantially less separation, with a smaller absolute difference and a weaker overall signal (*p* = 0.002) (Supplemental Figure S2B). Consistent with the primary PSM findings, IPTW-weighted Cox regression in Arm 1 demonstrated that omission of RT remained independently associated with increased mortality (HR, 3.26; 95% CI, 2.52–4.21; *p* < 0.001), even after adjustment for age and Charlson–Deyo comorbidity index (Supplemental Table S1). Age and comorbidity burden also remained significant predictors of mortality in Arm 1 (both *p* < 0.001). In Arm 2, the IPTW Cox estimate for RT omission was attenuated and not statistically significant (HR, 1.78; 95% CI, 0.80–3.95; *p* = 0.16), whereas age (*p* < 0.001) and comorbidity burden (*p* < 0.001) were significantly associated with worse survival (Supplemental Table S1), suggesting that the overall survival signal related to RT omission in this cohort was comparatively weaker and more uncertain. Finally, IPTW-based RMST analyses further supported a durable survival advantage associated with receipt of RT in Arm 1, with increasingly positive RMST differences over longer truncation horizons, while Arm 2 demonstrated smaller and less stable RMST differences over time, with wider uncertainty near the tail of follow-up (Supplemental Figure S3). Collectively, these IPTW results reinforce the robustness of the Arm 1 finding that RT omission is associated with poorer overall survival, while highlighting the limited precision and attenuated effect estimates in Arm 2.

Across analytic approaches, omission of RT was consistently associated with inferior survival in the adjuvant cohort (Arm 1), with concordant separation on Kaplan–Meier curves, elevated hazard ratios in Cox models, and increasing RMST differences over time. In the neoadjuvant pCR cohort (Arm 2), effect estimates generally favored RT but were limited by the small number of deaths and corresponding uncertainty, and results should be interpreted as hypothesis-generating.

## 4. Discussion

This NCDB analysis provides real-world evidence regarding the association between adjuvant RT and overall survival in an early-stage HER2-positive population selected to match the eligibility framework of NRG-BR008 (HERO) [8,9,10]. These findings are consistent with the established role of adjuvant radiation in improving local control and overall survival following breast-conserving surgery, including in HER2-positive disease. Current NCCN and ESMO guidelines continue to recommend adjuvant whole-breast RT after BCS for invasive breast cancer, including HER2-positive disease, and they do not endorse routine RT omission for HER2-positive tumors outside a clinical trial setting [4]. In this context, our HERO-aligned NCDB analysis provides pragmatic, practice-relevant evidence: in patients treated with adjuvant systemic therapy (Arm 1), RT omission was associated with significantly inferior OS, supporting continued guideline-concordant RT in routine care while prospective de-escalation data remain unavailable. In the neoadjuvant pCR cohort (Arm 2), effect estimates favored RT but remained imprecise due to sparse events, reflecting uncertainty rather than equivalence and reinforcing that RT omission should generally be reserved for clinical trials or exceptional circumstances after multidisciplinary discussion [8,9,10]. More recent population-based studies have also shown persistent reductions in recurrence and mortality with adjuvant radiation, despite the substantial improvements in systemic therapy outcomes afforded by trastuzumab and related agents [5,14,15,16,17]. The present results extend this evidence, showing adjuvant radiation treatment to be associated with clinically and statistically meaningful survival benefits in patients with early-stage breast cancer.

In the adjuvant systemic treatment cohort, RT was associated with a notable survival benefit, including an 25.8% absolute improvement in 10-year OS (94.3% vs. 68.5%), an approximate 79% reduction in mortality risk (HR≈0.21), and an RMST advantage of over 12.6 months at 10 years. These findings demonstrate a strong association between RT receipt and improved OS in the adjuvant cohort; however, given the magnitude of the observed association, attention to residual confounding and treatment selection bias is warranted. By contrast, the neoadjuvant cohort demonstrated a smaller, nonsignificant survival benefit, with a 5-year OS difference of ~6.5% and ~3.4× the mortality risk in patients who omitted RT. RMST analysis, which is less sensitive to proportional hazards assumptions, identified small but statistically significant survival advantages favoring radiation, +1.8 months at 5 years, and +3.9 months at 107 months. Taken together, these findings suggest that radiation may continue to provide incremental benefit in the neoadjuvant setting, but the magnitude of effect is likely attenuated compared with the adjuvant context, and larger studies with longer follow-up are needed to clarify its role.

The observed hazard ratio of 4.78 for RT omission in the adjuvant exceeds effect sizes reported in randomized trials of breast-conserving therapy. For context, the Early Breast Cancer Trialists’ Collaborative Group (EBCTCGs) meta-analysis demonstrated that adjuvant RT after breast-conserving surgery reduces the 15-year risk of breast cancer death by approximately 4–5%, corresponding to substantially more modest relative effect sizes [1]. The magnitude observed in the present analysis therefore strongly suggests the influence of residual confounding, likely reflecting confounding by indication or treatment selection bias. In routine clinical practice, patients in whom RT is omitted are unlikely to be exchangeable with those who receive RT and may differ systematically in ways not captured by the NCDB, including subtle decrements in functional or performance status, clinician assessment of biologic risk, anticipated tolerance of treatment, or competing non-oncologic health risks. As a result, the estimated hazard ratio should be interpreted as an upper bound of association within a heterogeneous, real-world population rather than as a precise estimate of RT’s isolated biological effect. Additionally, because the NCDB does not provide cause of death, we cannot determine whether the observed OS separation reflects differences in breast cancer mortality versus differential non-cancer mortality; residual frailty and competing-risk differences may therefore partially contribute. Importantly, while the extreme magnitude cautions against causal interpretation, the consistent direction and statistical significance of the association across analytic approaches remain informative and reinforce the need for careful evaluation of RT omission strategies outside the context of prospective trials.

Our results are broadly consistent with prior database studies examining RT in HER2-positive breast cancer [15,16,17]. Using a SEER-based analysis, Yang et al. [14] reported a significant survival advantage for patients receiving RT (HR = 0.45), although our observed effect size was even larger (HR ≈ 0.21). This difference may be attributable to stricter eligibility criteria aligned with HERO and systemic therapy sequence arm stratification. Similar findings have been reported by Bazan et al. in patients who received adjuvant systemic therapy [16] as well as patients who achieved complete pathologic response with neoadjuvant systemic therapy [17].

Biologically, the benefit of RT may extend beyond local control, potentially preventing the seeding of distant metastases by resistant tumor clones [18]. Even when absolute rates of local recurrence are low, this residual risk may still translate into meaningful differences in long-term survival. The synergy between effective systemic therapy and robust local control supports a comprehensive, multimodality approach, particularly for patients who are otherwise healthy and have a low comorbidity burden.

Importantly, advances in radiation delivery, including intensity-modulated RT, deep inspiration breath-hold (DIBH) [19], prone positioning [20], RT boosting [21], and hypofractionation [22,23], have substantially reduced the toxicity burden of treatment. These modern techniques mitigate concerns about long-term side effects, particularly cardiopulmonary exposure for left-sided tumors, further strengthening the case for continued RT use in appropriate patients.

This study has several limitations. First, the hospital-based nature of the NCDB, which captures approximately 70% of newly diagnosed cancers in the United States through Commission on Cancer (CoC)-accredited programs [11], may limit the generalizability of our findings to patients treated outside such centers. This is particularly relevant for evaluating radiation therapy access, as demonstrated by SEER-Medicare analyses showing rural patients with breast cancer traveled nearly three times farther for radiation than urban patients (40.8 vs. 15.4 miles) [24], and by population-based registry data indicating lower receipt of recommended RT among rural vs. urban patients (62.1% vs. 69.1%) [25]. Furthermore, disparities in systemic therapy completion can compound these access issues; for example, among women initiating adjuvant trastuzumab for early-stage HER2-positive disease at NCCN centers, Black women were less likely than White women to complete >270 days of therapy (73% vs. 87%) [26]. In addition, the NCDB does not specify HER2-directed agent(s), treatment duration, sequencing, or adherence, precluding differentiation between de-escalated trastuzumab-based regimens and intensified approaches such as dual HER2 blockade with pertuzumab or post-neoadjuvant adjuvant T-DM1 for residual disease, and it cannot capture variation in trastuzumab duration studied in randomized trials [6,27,28,29,30]. Given that our matched cohort was predominantly urban (80.6%) and privately insured (45.4%), the observed survival differences associated with RT likely reflect outcomes in comparatively well-resourced settings. Therefore, these results may not generalize to underserved populations.

Second, to align with the HERO (NRG-BR008) trial framework and reduce biological heterogeneity, we restricted our analysis to patients aged ≥ 40 years [6,7,8]. This threshold is justified by evidence that breast cancer diagnosed before age 40 represents a distinct biological entity, characterized by different biomarker distributions and higher proliferative activity (e.g., Ki-67), the latter of which has weaker prognostic value in this younger group [31]. Furthermore, young-onset breast cancer is enriched for germline BRCA1/2 pathogenic variants (12% in the prospective POSH cohort), which influence locoregional management strategies and competing risks [32]. It is important to note, however, that in the context of contemporary anti-HER2 regimens, young age (≤40 years) was not independently prognostic for invasive disease-free survival in the randomized APHINITY trial (adjusted HR, 1.07; 95% CI, 0.84–1.35), suggesting effective systemic therapy may mitigate age-associated risk [33]. While this age restriction improved cohort homogeneity and propensity score balance for our comparative analysis, it limits the generalizability of our findings to patients under 40, a population that merits dedicated study.

Third, there are limitations regarding the NCDB relevant to the interpretation of our data. As a retrospective, registry-based analysis, residual confounding from unmeasured factors (e.g., margin status, systemic therapy adherence, Ki-67 status, and social determinants of health) cannot be excluded. In particular, the NCDB does not capture HER2-directed agent selection, duration, or adherence. It also lacks granular RT treatment details (target volumes/fields, fractionation, dose constraints); while newer molecular biomarkers are currently being collected, inclusion in prior years is limited and incomplete, limiting adjustment for these potential confounders. Furthermore, the NCDB does not capture recurrence or cause-specific mortality, preventing separation of locoregional failure from distant relapse. Consequently, our findings represent a real-world association pertinent to clinical practice, where treatment variables are interdependent, but they cannot isolate the effect of RT independent of systemic therapy details.

Finally, although propensity score matching and survival-time left truncation were used to reduce selection and immortal time biases, these methods necessarily reduced the number of analyzable events and limited precision, most notably in the neoadjuvant cohort. This is emphasized with the reported post hoc (“observed”) power to demonstrate the confidence interval and reflect event sparsity. The wide confidence interval in Arm 2 indicates substantial uncertainty, and effect estimates in sparse-event settings may be inflated. These findings should therefore be interpreted cautiously and considered hypothesis-generating.

Our study findings suggest that adjuvant radiation therapy continues to show an association with meaningful survival benefit in early-stage HER2-positive breast cancer, even in the era of highly effective systemic therapy. Until results from prospective de-escalation trials such as HERO become available, the evidence presented here supports maintaining radiation therapy as a standard component of multimodality treatment for early-stage HER2-positive breast cancer. Omission of radiation should be considered only in highly selected patients and ideally within the context of a clinical trial.

## 5. Conclusions

In this propensity score-matched analysis of patients with early-stage HER2-positive breast cancer in the National Cancer Database, omission of radiation was associated with substantially inferior overall survival among patients treated with adjuvant systemic therapy. In the neoadjuvant cohort, survival differences between treatment groups were smaller and not statistically significant, although restricted mean survival time analyses suggested a modest advantage with radiation. These results reinforce the importance of adjuvant radiation therapy as a standard component of breast-conserving treatment and highlight risks of radiation omission outside clinical trials. Ongoing prospective de-escalation studies will be essential to determine whether radiation can be safely withheld in select patients achieving exceptional response to systemic therapy.

## Figures and Tables

**Table 1:** Baseline characteristics of matched cohorts. Baseline demographic and clinical characteristics of patients with early-stage HER2-positive breast cancer stratified by treatment sequence (Arm 1: adjuvant systemic therapy; Arm 2: neoadjuvant systemic therapy) and receipt of radiation therapy (RT). Continuous variables are shown as mean (standard deviation); categorical variables as number (percentage). SMD = standardized mean difference. Stratification variables (age ≥ 60 years, tumor size > 1 cm, ER-positive status) were balanced by design. Survival time was left-truncated at 6 months to mitigate immortal time bias.

Baseline Characteristics of Matched Cohorts by Trial Arm						
	Arm 1 Cohort Adjuvant Systemic Therapy			Arm 2 Cohort Neoadjuvant Systemic Therapy		
Characteristic	RT (*N* = 409)	No RT (*N* = 409)	SMD	RT (*N* = 88)	No RT (*N* = 88)	SMD
**Age, mean (SD)**	63.39 (10.68)	63.45 (10.68)	0.006	60.14 (10.37)	60.24 (10.59)	0.010
**Sex, Female (%)**	409 (100.0)	407 (99.5)	0.099	88 (100.0)	88 (100.0)	<0.001
**Race (%)**			0.076			0.061
White	344 (84.1)	346 (84.6)		65 (73.9)	67 (76.1)	
Black	49 (12.0)	47 (11.5)		16 (18.2)	14 (15.9)	
Asian	12 (2.9)	12 (2.9)		5 (5.7)	5 (5.7)	
American Indian or Alaska Native	1 (0.2)	0 (0.0)		0 (0.0)	0 (0.0)	
Native Hawaiian or Pacific Islander	0 (0.0)	0 (0.0)		0 (0.0)	0 (0.0)	
Unknown	3 (0.7)	4 (1.0)		2 (2.3)	2 (2.3)	
**Ethnicity, Hispanic (%)**	27 (6.6)	25 (6.1)	0.020	8 (9.1)	9 (10.2)	0.038
**Comorbidity index, mean (SD)**	0.22 (0.51)	0.25 (0.54)	0.019	0.23 (0.50)	0.23 (0.52)	<0.001
**Insurance (%)**			0.058			0.202
Uninsured	4 (1.0)	4 (1.0)		3 (3.4)	2 (2.3)	
Private	166 (40.6)	168 (41.1)		49 (55.7)	48 (54.5)	
Medicaid	19 (4.6)	18 (4.4)		6 (6.8)	7 (8.0)	
Medicare	213 (52.1)	209 (51.1)		27 (30.7)	30 (34.1)	
Other Government	3 (0.7)	5 (1.2)		1 (1.1)	0 (0.0)	
Unknown	4 (1.0)	5 (1.2)		2 (2.3)	1 (1.1)	
**Urban–Rural Influence (%)**			0.058			0.198
Urban	347 (84.8)	348 (85.3)		77 (87.5)	88 (92.0)	
Metro	47 (11.5)	49 (12.0)		9 (10.2)	6 (6.8)	
Rural	3 (0.7)	2 (0.5)		1 (1.1)	1 (1.1)	
Unknown	12 (2.9)	9 (2.2)		1 (1.1)	0 (0.0)	
**Year of Diagnosis (%)**			0.025			0.052
2007–2013	79 (19.3)	83 (20.3)		5 (5.7)	4 (4.5)	
2014–2017	330 (80.7)	326 (79.7)		83 (94.3)	84 (95.5)	
**Tumor Grade (%)**			0.061			0.099
Low (I–II)	196 (47.9)	196 (47.9)		30 (34.1)	29 (33.0)	
High (III–IV)	204 (49.9)	200 (48.9)		54 (61.4)	53 (60.2)	
Unknown	9 (2.2)	13 (3.2)		4 (4.5)	6 (6.8)	
**Lymphovascular Invasion (%)**			0.032			0.099
Absent	321 (78.5)	316 (77.3)		56 (63.6)	53 (60.2)	
Present	40 (9.8)	41 (10.0)		5 (5.7)	7 (8.0)	
Unknown	48 (11.7)	52 (12.7)		27 (30.7)	28 (31.8)	
**Stratification Variables (%)**						
Age ≥ 60	264 (64.5)	264 (64.5)	<0.001	43 (48.9)	43 (48.9)	<0.001
Size >1 cm	266 (65.0)	266 (65.0)	<0.001	83 (94.3)	83 (94.3)	<0.001
ER positive	315 (77.0)	315 (77.0)	<0.001	44 (50.0)	44 (50.0)	<0.001

**Table 2:** Multivariate Cox proportional hazards regression (PSM-matched). PSM-matched multivariable Cox proportional hazards regression for overall survival in matched cohorts. Hazard ratios reflect mortality risk associated with omission of radiation therapy (no RT, experimental group) compared with receipt of radiation (RT, control group). Models were adjusted for Charlson–Deyo comorbidity index and age and stratified by tumor size (≤1 cm vs. >1 cm) and ER status (positive vs. negative) in accordance with the HERO trial protocol. A hazard ratio >1 indicates increased mortality risk. Results are reported separately for Arm 1 (adjuvant systemic therapy cohort) and Arm 2 (neoadjuvant systemic therapy cohort), with corresponding 95% confidence intervals and *p* values.

Multivariate Cox Proportional Hazards Regression (PSM Model)				
**Arm 1: Adjuvant Cohort**	**Hazard Ratio (95% CI)**	**Standard Error**	**Z score**	***p* value**
No Radiation (Experimental Group)	4.777 (2.844–8.024)	0.2624	5.91	<0.001
Charlson–Deyo Comorbidity Index	1.439 (1.085–1.908)	0.1544	2.53	0.01
Age (Years)	1.075 (1.051–1.101)	0.0126	6.16	<0.001
**Arm 2: Neoadjuvant Cohort**	**Hazard Ratio (95% CI)**	**Standard Error**	**Z score**	***p* value**
No Radiation (Experimental Group)	3.402 (0.823–14.054)	0.7149	1.69	0.09
Charlson–Deyo Comorbidity Index	2.002 (0.868–4.615)	0.4459	1.63	0.10
Age (Years)	1.099 (1.038–1.164)	0.0355	3.26	<0.01

**Table 3:** Post hoc power and minimum detectable effect size (Schoenfeld approximation). Post hoc power was estimated using Schoenfeld’s method based on the observed number of deaths and treatment allocation in the propensity score-matched cohorts. Power is shown for selected hazard ratios comparing receipt of adjuvant radiation therapy (RT) vs. omission of radiation therapy (no RT), assuming a two-sided α = 0.05. Underlined are observed hazard ratios using propensity score matched-cohorts. Minimum detectable effect sizes represent the hazard ratio required to achieve 80% power given the observed event counts.

Power Analysis for PSM-Weighted Cohorts				
Arm	Events (RT vs. No RT)	Allocation (1:1)	Hazard Ratio	Power
1	18 vs. 76	0.5	1.2	14.1%
**1**	18 vs. 76	0.5	1.3	24.6%
1	18 vs. 76	0.5	1.5	50.2%
1	18 vs. 76	0.5	2	91.9%
1	18 vs. 76	0.5	3	100%
1	18 vs. 76	0.5	4.78	100%
2	3 vs. 7	0.5	1.2	4.7%
2	3 vs. 7	0.5	1.3	6.1%
2	3 vs. 7	0.5	1.5	9.3%
2	3 vs. 7	0.5	2	19.4%
2	3 vs. 7	0.5	3	41.2%
2	3 vs. 7	0.5	3.40	49.0%
2	3 vs. 7	0.5	4	59.2%
2	3 vs. 7	0.5	5	72.1%
2	3 vs. 7	0.5	6	80.9%

## Data Availability

The data that support the findings of this study are available from the NCDB, but restrictions apply to the availability of these data, which were used under license for the current study, and so are not publicly available. Data are, however, available from the authors upon reasonable request and with permission of the NCDB.

## Supplementary Materials

The following supporting information can be downloaded at https://www.mdpi.com/article/10.3390/cancers18030352/s1, Figure S1: Covariate Balance Using Inverse Probability of Treatment Weighting. Figure S2: Kaplan-Meier Overall Survival Curves (IPTW-Matched). Figure S3: Restricted Mean Survival Time Analysis (IPTW-Matched). Table S1: Multivariate Cox Proportional Hazards Regression (IPTW-Matched).

## Abbreviations

BCS	breast-conserving surgery
ER	estrogen receptor
HR	hazard ratio
IPTW	inverse probability of treatment weighting
NCDB	National Cancer Database
OS	overall survival
PSM	propensity score matching
RMST	restricted mean survival time
RT	radiation therapy
