# Supplementary material for: Evaluating Adjuvant Radiation Therapy Survival Benefit in Early-Stage HER2-Positive Invasive Breast Cancer Following Breast-Conserving Surgery: A National Cohort Aligned with NRG-BR008 HERO Trial
Source: Cancers (Basel). 2026 Jan 23;18(3):352. doi: 10.3390/cancers18030352 (PMC12896820; doi:10.3390/cancers18030352)
Supplement: Supplementary file 1 [file cancers-18-00352-s001.zip › cancers-4062192-supplementary.pdf]

Supplemental

The Supplemental Materials provide sensitivity and robustness analyses to complement the primary propensity score–matched (PSM) findings. Supplemental Figures S1–S3 summarize results from inverse probability of treatment weighting (IPTW), including covariate balance diagnostics (Supplemental Figure S1), weighted Kaplan-Meier overall survival curves with risk tables (Supplemental Figure S2), and restricted mean survival time (RMST) differences over time under the IPTW-adjusted framework (Supplemental Figure S3). Supplemental Table S1 reports multivariable Cox proportional hazards regression estimates from the IPTW-weighted cohorts for both the adjuvant (Arm 1) and neoadjuvant (Arm 2) systemic therapy analyses. Collectively, these supplemental analyses evaluate whether the direction and magnitude of associations between radiation omission and survival outcomes remain consistent under an alternative causal inference approach.

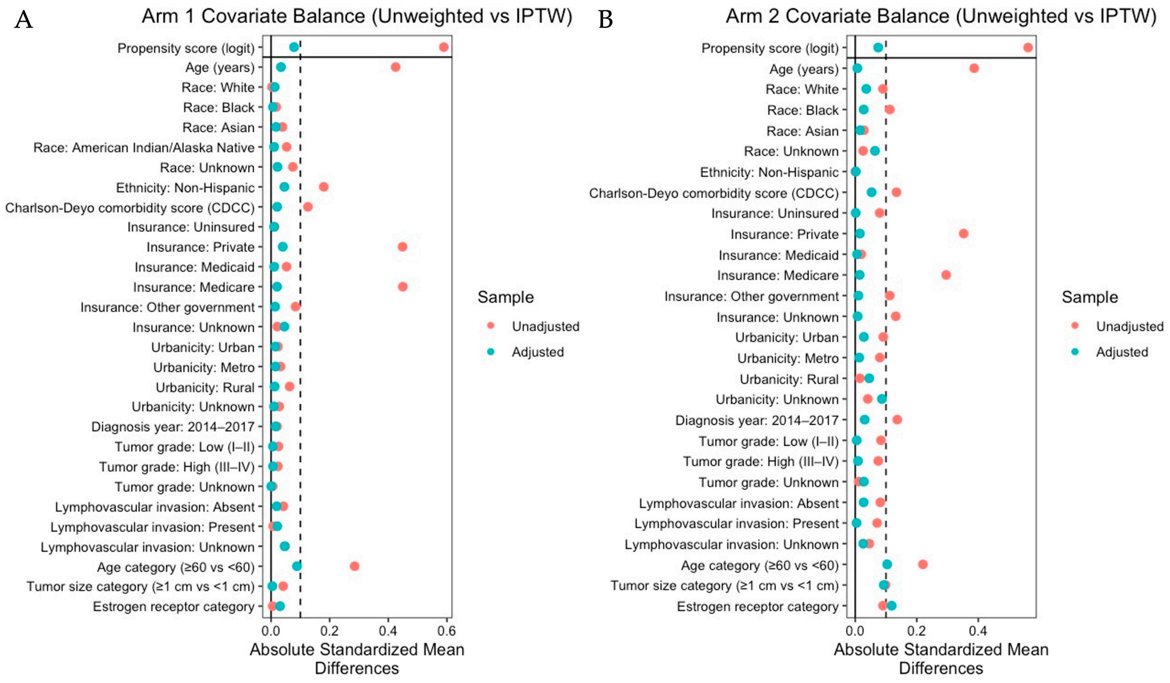

Figure S1. Covariate Balance Using Inverse Probability of Treatment Weighting. Love plots depicting absolute standardized mean differences (ASMDs) for baseline covariates comparing patients who received adjuvant radiation therapy (RT) vs those who omitted RT (No RT) before weighting (unadjusted; red) and after inverse probability of treatment weighting (IPTW-adjusted; teal). Panel A shows covariate balance for Arm 1 (Adjuvant systemic therapy cohort), and Panel B shows covariate balance for Arm 2 (Neoadjuvant systemic therapy cohort). The vertical dashed line indicates the balance threshold (ASMD = 0.10); values below this threshold are generally interpreted as indicating adequate post-matching balance between treatment groups.

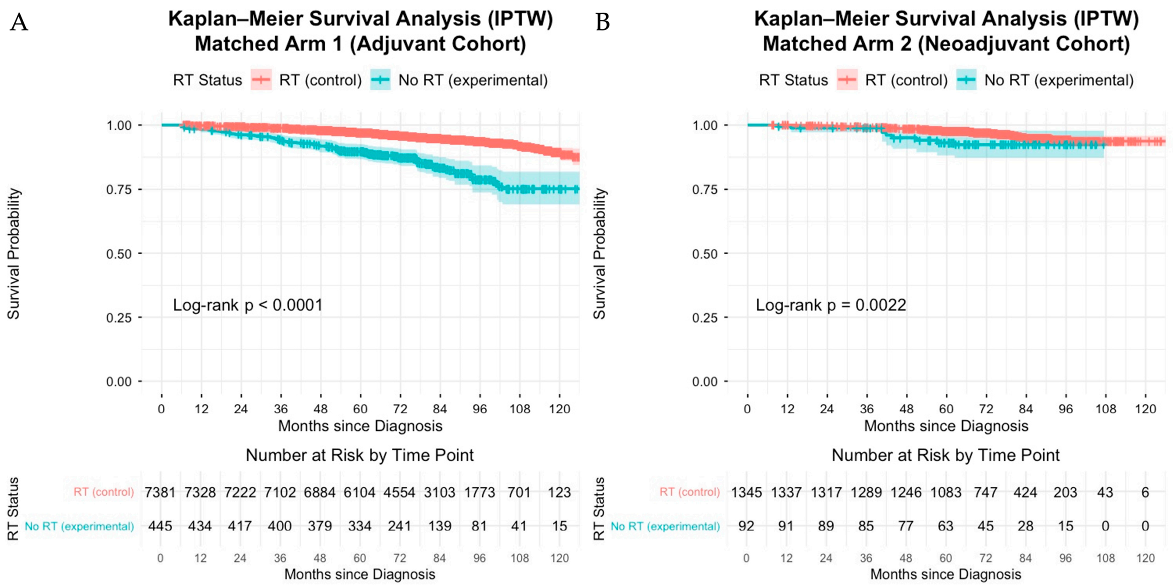

Figure S2. Kaplan-Meier Overall Survival Curves (IPTW-Matched). Kaplan-Meier estimates of overall survival (OS) comparing IPTW-matched patients treated with adjuvant radiation therapy (RT) vs no RT, stratified by treatment sequence: Arm 1 (Adjuvant systemic therapy cohort), Panel A, and Arm 2 (Neoadjuvant systemic therapy cohort), Panel B. Survival time was left-truncated at 6 months to mitigate immortal time bias. P values reflect log-rank tests stratified by age, tumor size, and estrogen receptor status. Shaded regions indicate 95% confidence intervals.

Table S1. Multivariate Cox Proportional Hazards Regression (IPTW-Matched). IPTW-matched multivariable Cox proportional hazards regression for overall survival in matched cohorts. Hazard ratios reflect mortality risk associated with omission of radiation therapy (no RT, experimental group) compared with receipt of radiation (RT, control group). Models were adjusted for Charlson-Deyo comorbidity index and age and stratified by tumor size (≤1 cm vs >1 cm) and ER status (positive vs negative) in accordance with the HERO trial protocol. A hazard ratio >1 indicates increased mortality risk. Results are reported separately for Arm 1 (Adjuvant systemic therapy cohort) and Arm 2 (Neoadjuvant systemic therapy cohort), with corresponding 95% confidence intervals and P values.

Multivariate Cox Proportional Hazards Regression (IPTW model)				
**Arm 1: Adjuvant Cohort**	**Hazard ratio (95% CI)**	**Standard error**	**Z score**	**P value**
No Radiation (Experimental Group)	3.257 (2.518–4.213)	0.1321	8.99	<.001
Charlson-Deyo Comorbidity Index	1.549 (1.349–1.778)	0.0666	6.21	<.001
Age (years)	1.078 (1.067–1.090)	0.0053	13.83	<.001
**Arm 2: Neoadjuvant Cohort**	**Hazard ratio (95% CI)**	**Standard error**	**Z score**	**P value**
No Radiation (Experimental Group)	1.778 (0.801–3.950)	0.4286	1.41	.16
Charlson-Deyo Comorbidity Index	1.924 (1.316–2.812)	0.1918	3.38	<.001
Age (years)	1.074 (1.049–1.101)	0.0143	5.80	<.001

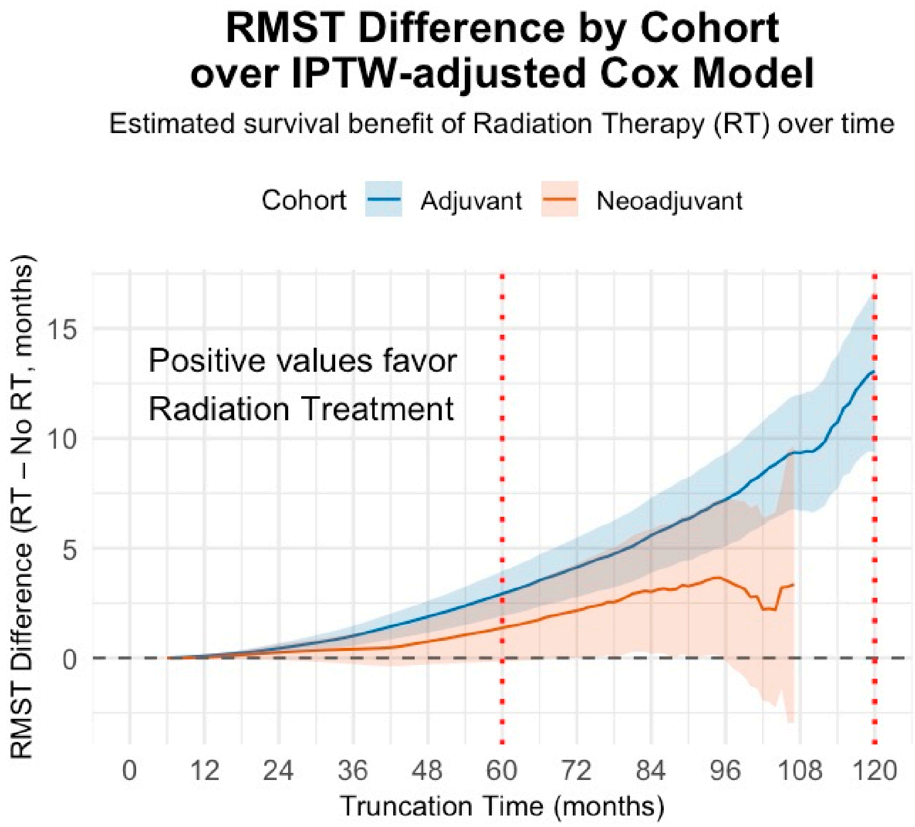

Figure S3. Restricted Mean Survival Time Analysis (IPTW-Matched). RMST difference curves showing the IPTW-matched absolute survival benefit of radiation therapy (RT) minus no RT across increasing follow-up durations stratified by treatment sequencing cohort (Arm 1 Adjuvant systemic therapy cohort; Arm 2 Neoadjuvant systemic therapy cohort). Positive RMST differences indicate a longer average survival time associated with RT. Shaded bands represent 95% confidence intervals; vertical red dotted lines denote clinically relevant follow-up timepoints (5 and 10 years). Survival time was left-truncated at 6 months in both cohorts to mitigate immortal time bias.
